# Investigation of phytochemical profiling and biological activities of methanol extract from *Eryngium billardieri*: antimicrobial, antibiofilm, and anthelmintic properties

**DOI:** 10.3389/fpls.2025.1667335

**Published:** 2025-11-06

**Authors:** Mahdi Yaghoobi, Mahdi Moridi Farimani, Ajmal Khan, Mojtaba Asadollahi, Marzieh Omrani, Walter Luyten, Haibo Hu

**Affiliations:** 1 Department of Phytochemistry, Medicinal Plants and Drug Research Institute, Shahid Beheshti University, Evin, Tehran, Iran; 2 Animal Physiology and Neurobiology Section, Department of Biology, KU Leuven, Leuven, Belgium; 3 Leishmania Diagnostic & Drug Delivery Research Laboratory, University of Peshawar, Peshawar, Pakistan; 4 Department of Natural Sciences, Mid Sweden University, Sundsvall, Sweden; 5 Jiangxi Province Key Laboratory of Pharmacology of Traditional Chinese Medicine, National Engineering Research Center for Modernization of Traditional Chinese Medicine - Hakka Medical Resources Branch, School of Pharmacy, Gannan Medical University, Ganzhou, China

**Keywords:** *Eryngium billardieri*, antimicrobial, methanol extract, antihelmintic, antibiofilm, antifungal

## Abstract

The genus *Eryngium*, belonging to the Umbelliferae family, comprises flowering plants with various pharmacological activities, including anti-inflammatory and antidiabetic properties. However, many of these activities lack scientific evaluation. This study aimed to characterize the metabolites and evaluate the antihelmintic, antibacterial, and antibiofilm activities of a methanolic extract derived from the aerial parts of *Eryngium billardieri*. Metabolite characterization was conducted using LC-MS combined with a computer-assisted structure elucidation method. The extract was tested against six fungi, six Gram-positive bacteria, and nine Gram-negative bacteria, and a non-parasitic nematode (*Caenorhabditis elegans)*. A total of thirty-three compounds were identified, with the major constituents including isorhamnetin-3-O-glucoside, phytolaccagenin, terpinolene, 3,4-dimethoxybenzaldehyde, palmitic acid, isobornyl formate, isorhamnetin, and 1,4-dimethyl-7-(1-methylethenyl)-octahydroazulene. Across all tested concentrations, Gram-positive bacteria demonstrated greater sensitivity compared to Gram-negative bacteria, with *Staphylococcus aureus* and *Micrococcus luteus* showing the highest sensitivity (IC_50_ values of 57.47 µg/mL and 105.8 µg/mL, respectively). Among Gram-negative strains, only *Brevundimonas diminuta* exhibited sensitivity. In antifungal tests, six of seven yeast strains displayed sensitivity to the extract, with *Candida parapsilosis* and *Candida albicans* being particularly susceptible (IC_50_ values of 11.29 µg/mL and 63.29 µg/mL, respectively). The antibiofilm analysis demonstrated inhibitory effects within 24 hours after biofilm formation, with an IC_50_ of 6.3 µg/mL. Additionally, the antihelmintic assay revealed a mean inhibition rate of 97.7 ± 1.5 at 2.0 µg/mL. The results demonstrate that the extract effectively inhibited the tested bacteria, particularly against yeast strains. While the extract showed promising activity against a model nematode, further research is imperative to validate its anthelmintic efficacy against parasitic nematodes.

## 1 Introductions

Medicinal plants, renowned for their antimicrobial, anticancer, anti-inflammatory, and diverse pharmacological properties, have been employed globally for millennia (Perumal Samy and Gopalakrishnakone, 2010; Wang et al., 2014; Sadeghi et al., 2022). However, with the advent of antibiotics and synthetic chemical drugs in the 20^th^ century, their use diminished, accompanied by a decline in scientific research into their effects. This shift has had significant repercussions for both human and animal health (Grenni et al., 2018). The inappropriate and widespread use of antibiotics has led to increasing resistance problems. In response, the European Union implemented regulations in 2006 to limit the use of antibiotics and other chemicals, aiming to curb the spread of antibiotic resistance among human pathogens. Consequently, efforts have intensified to explore plants or plant-derived extracts as natural alternatives (Alamgir, 2017).

The rise of resistance to synthetic drugs poses a significant challenge to public health (McEwen and Collignon, 2018). While various chemical drugs with distinct structures and mechanisms are available for treating helminthic, bacterial, and fungal infections, resistance often results in persistent, acute, or recurrent diseases (Manandhar et al., 2019). Prolonged drug usage has, in some cases, led to adverse side effects, restricting their therapeutic potential. Resistant microorganisms pose a substantial risk to human populations, animals, and plants (Manandhar et al., 2019; Saad et al., 2023). Presently, researchers are striving to optimize the use of chemical drugs, prevent diseases, and develop new, less toxic compounds with fewer side effects (Carracedo−Reboredo et al., 2021). Remarkably, although most drugs today are synthetic, at least one-third of these agents originate from plants or are derived from plant extracts. For millennia, plants have served as remedies, immune system enhancers, and agents against cancer and infections, and they remain invaluable sources in the quest for effective and safe therapeutic solutions (Romão et al., 2013; Mousavi et al., 2025).

**Figure 1:**
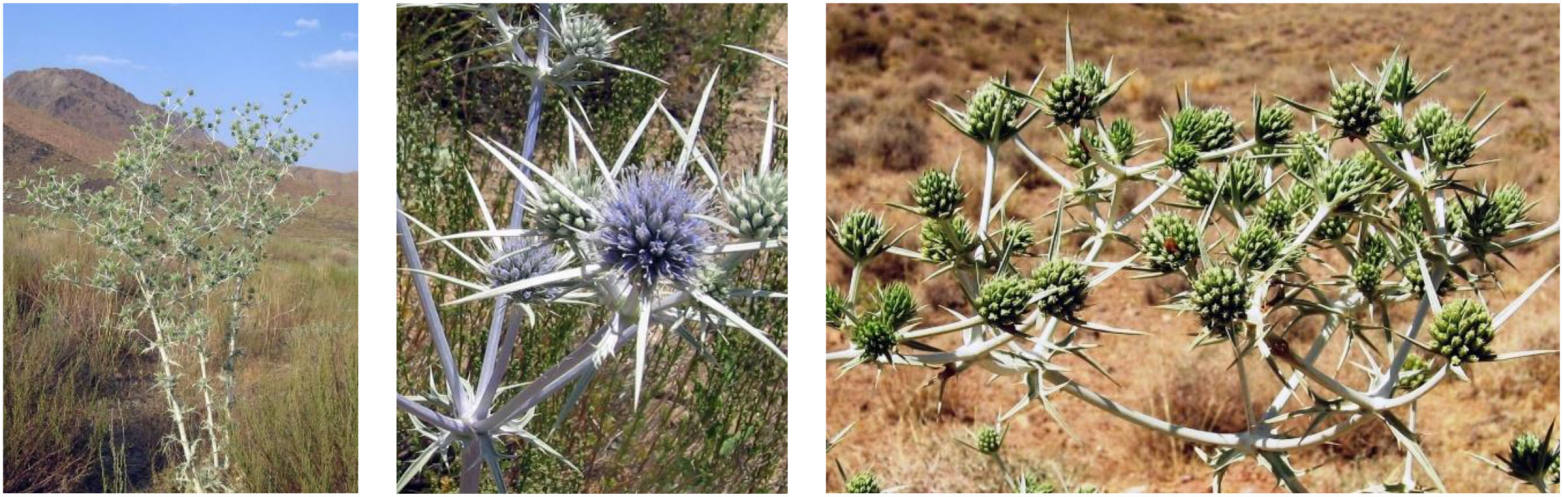
*Eryngium billardieri* F. Delarche.

*Eryngium billardieri* F. Delarche. (**Figure 1**), a member of the Umbelliferae family, is native to the Iran–Turonian floristic region, the species inhabits steppe ecosystems from plains to montane zones, favoring rocky, well-drained, nutrient-poor soils and full sun. It is intolerant to prolonged soil saturation and commonly colonizes disturbed habitats, including overgrazed rangelands, with populations often increasing after fire events (Bashari et al., 2025). *E. billardieri* has long been employed in traditional medicine to treat various inflammatory disorders (Osqueei et al., 2023). In Iranian traditional medicine, the aerial parts of *E. billardieri* have been used to address a wide range of conditions, including goiter (Kremer et al., 2021), lymphedema, inflammatory disorders (Daneshzadeh et al., 2020), rheumatism, hyperglycemia (Osqueei et al., 2023), urinary infections, and wound healing (Küpeli et al., 2006; Sepanlou et al., 2019). Notably, previous reports have highlighted the anti-inflammatory and anti-hyperglycemic effects of *E. billardieri*’s roots and aerial parts (Kremer et al., 2021). Furthermore, the cytotoxicity of *E. billardieri* extracts against PANC-1 cancer cells has been evaluated (Hasanbeiglu et al., 2022). Recent investigations have shown that both essential oil and solvent extracts of *E. billardieri* exhibit significant antibacterial activity. Hajian-Maleki and Shams-Bakhsh (2023) demonstrated that the plant’s essential oil exerts strong inhibitory effects against several Gram-positive and Gram-negative bacteria, producing inhibition zones of approximately 8–21 mm and minimum inhibitory concentrations ranging from 0.67 g L^−1^ to 34.17 g L^−1^. Gas chromatography–mass spectrometry (GC–MS) analysis identified 34 constituents accounting for over 95% of the total oil composition, with n-hexadecanoic acid, 2-pentadecanone, and cinnamyl tiglate among the major bioactive compounds (Hajian-Maleki and Shams-Bakhsh, 2023). Similarly, Farhan et al. (2012) reported antibacterial effects of crude *E. billardieri* extracts, with variations depending on solvent type and bacterial strain (Farhan et al., 2012). In another study, Allafchian et al. (2022) utilized *E. billardieri* extract in the green synthesis of silver nanoparticles and observed enhanced antimicrobial activity of the resulting nanocomposites against multiple bacterial species (Allafchian et al., 2022).

The growing resistance to current therapeutic agents for human and animal diseases underscores the urgent need for novel treatments (Küpeli et al., 2006; Rauf et al., 2021; Zargar Zarin et al., 2025). Plant-based drugs have garnered widespread attention (Lu et al., 2020; Shawky et al., 2021) due to their perceived safety and reliability as alternatives to expensive synthetic drugs (Ernst, 2007). Consequently, medicinal plants are subjected to extensive screening for potential biological activities (Romano et al., 2021). Although some plants have been extensively used by traditional healers as antiparasitic and antimicrobial agents, their efficacy under experimental conditions remains largely unverified (Zulhendri et al., 2021). Traditional Iranian medicinal plants, including *E. billardieri*, may offer more effective treatments for infections caused by parasites and microbes (Amiri et al., 2021; Rashidipour et al., 2022; Sadeghi et al., 2023; Tabefam et al., 2018). In continuing our research on Iranian medicinal plant, here in, *E. billardieri* is investigated for its antihelmintic, antibacterial, and antifungal properties, aiming to scientifically validate its traditional use by the local population of Iran.

## 2 Material and methods

### 2.1 Chemical reagents

Various chemical reagents and solvents, including acetonitrile, dimethyl sulfoxide (DMSO), ethyl acetate, formic acid, *n*-hexane, trifluoroacetic acid, and methanol, were purchased from Chem-Lab NV (Zedelgem, Belgium). Miconazole (200 mg/mL stock) served as the positive control for antifungal activity. Ciprofloxacin (100 μg/mL) and levamisole, both purchased from Sigma-Aldrich, were used as positive controls for antibacterial and anthelmintic activities. Resazurin salt was sourced from Acros Organics in Geel, Belgium. A Milli-Q system (Millipore, Bedford, MA, USA) was used to prepare deionized water.

### 2.2 Plant material and extraction

*Eryngium billardieri* F. Delarche. was collected in Shiraz, Fars Province, Iran, in May 2019. The plant was identified by Dr. Mojtaba Asadollahi, a botanist, and a voucher specimen (MPH-2698) was deposited at the herbarium of the Medicinal Plants and Drug Research Institute, Shahid Beheshti University, Tehran, Iran. The aerial parts (1.5 kg) were washed, dried in the laboratory away from direct sunlight, ground into powder, and subjected to methanol extraction three times (7 L×3, successively) at room temperature, with each cycle lasting 72 hours. The solvent was removed under reduced pressure using rotary evaporation at 40 ^°^C. The drying process continued until a dry mass with a constant weight of 120 g was obtained. The extract was stored at 4 °C for further analysis.

### 2.3 Biological assay

#### 2.3.1 Antimicrobial assay

The extract was tested against six yeasts (*Candida albicans, Candida auris, Candida parapsilosis, Candida utilis, Candida glabrata, Saccharomyces cerevisiae*), six Gram-positive bacteria (*Staphylococcus aureus, Micrococcus luteus, Enterococcus faecalis, Streptococcus faecalis, Staphylococcus epidermidis, Listeria innocua)*, and nine Gram-negative bacteria (*Escherichia coli, Pseudomonas aeruginosa, Aeromonas hydrophila, Shigella flexneri, Shigella sonnei, Acinobacter baumanii, Enterobacter aerogenes, Brevundimonas diminuta, Salmonella enteritidis*). The test organisms used in this study were sourced from the American Type Culture Collection (ATCC) and are stored in our laboratory’s freezer/fridge for future use. The antimicrobial activity was assessed using a broth microdilution assay following the method described by Hu et al., 2023. Briefly, yeast cultures were grown in YPD medium (1% yeast extract, 2% peptone, and 2% dextrose), while bacterial cultures were grown in Mueller–Hinton (MH) medium (0.2% beef extract, 1.75% casamino acids, and 0.015% soluble starch). Under aseptic conditions, 5 μL of bacterial cultures (1×10^6^ CFU/mL) and 10 μL of yeast cultures (1×10^5^ CFU/mL) were inoculated in 96-well plates along with 10 μL of the test sample, solvent control (DMSO), and positive controls (miconazole at 200 μg/mL and ciprofloxacin at 100 μg/mL). The test organisms were adjusted to an optical density (OD) of 0.003 for bacteria and 0.001 for fungi and incubated at 37 °C for 20 hours. Miconazole (for fungi) and ciprofloxacin (for Gram-positive and Gram-negative bacteria) were included as positive controls (Hu et al., 2023). The inhibition values were calculated using the following **Equation 1**:

(1) $$\%\text{Inhibition}=\frac{\text{Sample OD value}-\text{Sample control}}{\text{Average OD of the controls}(\text{Solvent})}*100$$

To ensure reliability, all experiments were conducted in replicate. The IC_50_ values were determined through nonlinear least-squares sigmoid regression curve fitting. Additionally, serial dilution agar tests were conducted to determine the minimum bactericidal concentration of the extract (Hu et al., 2022).

#### 2.3.2 Antibiofilm test

The biofilm-forming strain was cultivated in Yeast Extract–Peptone (YPD) broth for *Candida* biofilms at 37 °C for 18–24 hours. The microorganism-containing culture was then centrifuged at 800 rpm for 2 minutes, and the supernatant was carefully discarded. One mL of RPMI-MOPS medium was added to the tube, and after gentle vortexing to ensure uniformity, the OD was measured and adjusted to 0.1 at 600 nm, corresponding approximately to 1 × 10^6^ CFU/mL of Candida albicans cells. A 100 μL aliquot of the *Candida* suspension in RPMI-MOPS was transferred into a 96-well plate and incubated at 37 °C for 90 minutes in a stationary incubator to facilitate the initial adhesion phase of biofilm formation. Following incubation, the medium was removed, and each well was washed three times with 100 μL phosphate-buffered saline (PBS) to eliminate non-adherent cells. Test samples and YPD media were added to each well. Additionally, DMSO control and positive control wells were included, with one well left empty for the subsequent resazurin control during staining. The plate was further incubated at 37 °C for 24 hours in a stationary incubator. Following this incubating, the medium was removed, and the wells were washed twice with PBS. Biofilm staining was conducted using 100 μL of resazurin dye (40 μg/mL) pre well. After one hour of incubation at 37 °C, fluorescence was measured using a FlexStation II spectrofluorometer (Molecular Devices, USA) with excitation (λ_ex_) and emission (λ_em_) wavelengths set at 535 nm and 590 nm, respectively. The following **Equation 2** was used to calculate the surviving biofilm percentage:

(2) $$\%\;\text{Surviving biofilm}=\frac{\text{Fluorescence readings of biofilm and samples}\;-\;\text{Alamar blue blank}}{\text{DMSO control}}\;*100$$

#### 2.3.3 Anthelmintic test

##### 2.3.3.1 Culture, maintenance and synchronization of *Caenorhabditis elegans*

*Caenorhabditis elegans* (*C. elegans*) strains were cultured on Petri dishes containing a lawn of *E. coli*. Synchronized populations were prepared using a modified alkaline bleaching method. In brief, eggs and adult worms were washed with S-basal medium and treated with a bleaching solution composed of 1 mL bleach and 0.5 mL of 5 M NaOH. The resulting suspension was washed several times using S-basal medium and incubated for 24 hours to obtain L1 larvae. These L1 larvae were then transferred onto a nematode growth media plate with an *E. coli* lawn and incubated at 20 °C until they reached the L4 larval stage. This developmental stage was used for the anthelminthic assay.

##### 2.3.3.2 Anthelmintic assay

The assay was performed following the method described by Cédric et al. (2023), with minor modifications. In summary, each well of a 96-well microplate was filled with 184 µL of *E. coli* culture (OD = 0.5 at 600 nm), followed by addition of synchronized *C. elegans* (L4 larvae) suspended in S-basal medium. Subsequently, 1 µL of plant extract was introduced into each well. Control wells contained 1 µL of DMSO as a solvent control and 50 µM levamisole as a positive control. The microplates were incubated at 20 °C for 16 hours in a WMicrotracker ONE system (Phylumtech), where worm movements were recorded every 30 minutes. The percentage inhibition of worm mobility was calculated using the following **Equation 3**:

(3) $$\%\text{Inhibition}=\frac{Worm\;mobility\;in\;treated\;wells-Mobility\;of\;worms\;in\;the\;negative\;control}{Mobility\;of\;worms\;in\;the\;negative\;control}\times 100$$

### 2.4 LC-MS analysis

The MS sample was redissolved in acetonitrile (MeCN) at a concentration of 0.1 mg/mL. For MS detection, 5 µL of a 20-fold dilution was injected. A Shimadzu LCMS-2020 system was employed for analysis, operating in full scan mode with a mass range of 100–1500 m/z in both positive and negative ion modes. The MS data were processed using various software tools including Xcalibur 4.2, Freestyle™ 1.5, ACD/MS Workbook Suite 2021 with MS Fragmenter, and ChromGenius.

### 2.5 Data processing and analysis

The dose-response data were analyzed using GraphPad Prism version 8.0 for Windows (GraphPad Software Inc., San Diego, CA, USA). To assess the *in vitro* activity, one-way analysis of variance (ANOVA) and Tukey’s multiple comparison test were employed. The 50% inhibitory concentrations (IC_50_) were determined by plotting concentration-response curves, where the logarithm of the concentration was plotted against the percentage inhibition. All the tests were repeated three times to ensure reliable data.

## 3 Results

### 3.1 Antimicrobial activity of the extract

#### 3.1.1 Effect of extract on gram-positive bacteria

**Figure 2:**
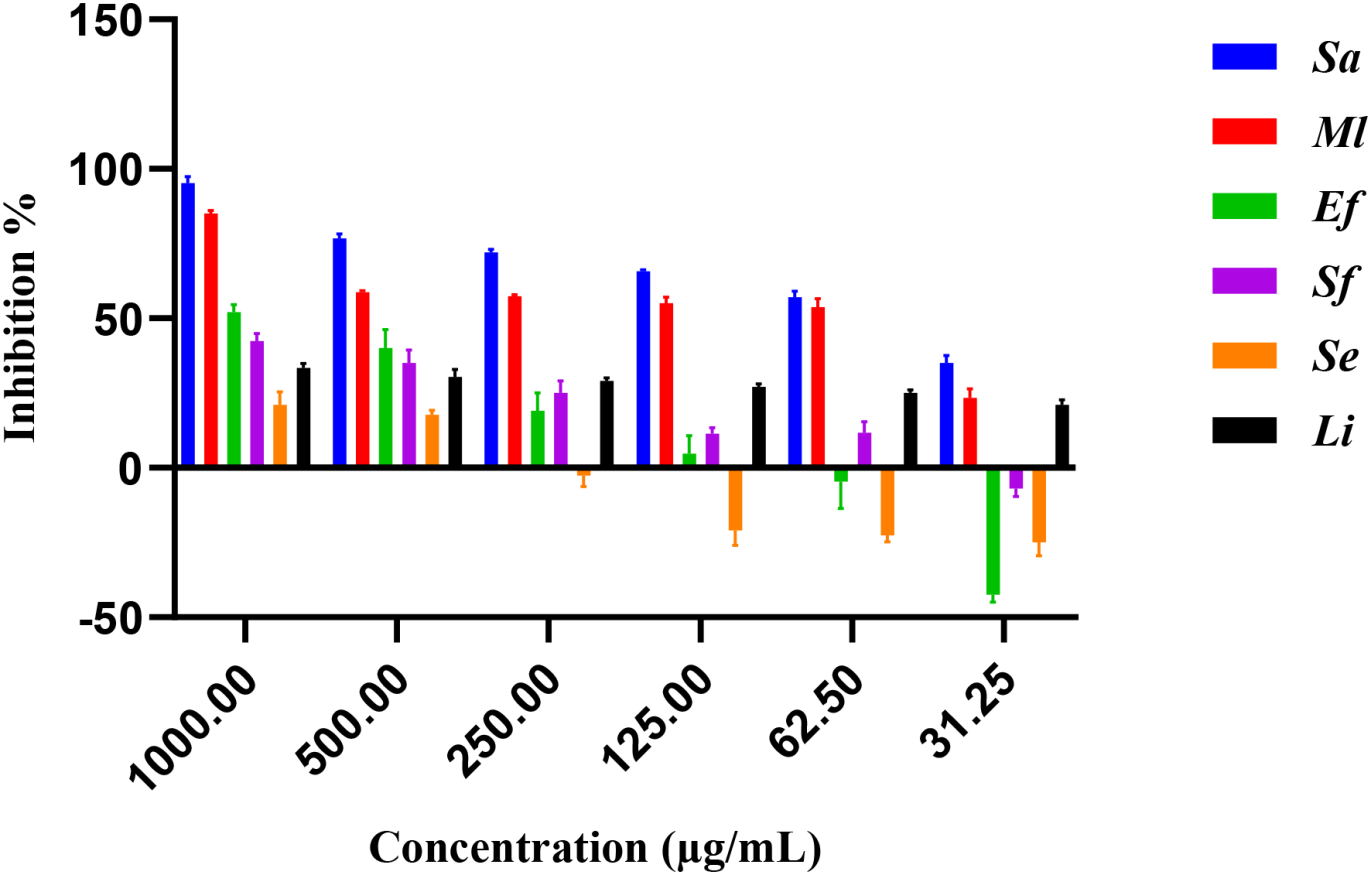
Percentage of inhibition of extract against gram-positive bacteria.

The antimicrobial activity of *E. billardieri* extract against Gram-positive bacteria is shown in **Figure 2**. The results indicate a concentration-dependent inhibition, with higher concentrations yielding greater effectiveness. Among the tested Gram-positive strains, *Staphylococcus aureus* and *Micrococcus luteus* demonstrated the highest sensitivity, with low IC_50_ values of 57.47 µg/mL and 105.8 µg/mL, respectively (**Table 1**). In contrast, *Enterococcus faecalis*, *Streptococcus faecalis*, and *Staphylococcus epidermis* showed relatively higher IC_50_ values (804 µg/mL, 1223 µg/mL, and 1892 µg/mL, respectively), indicating moderate resistance. *Listeria innocua* displayed the highest IC_50_ value (67.245 µg/mL), suggesting that it is the least susceptible Gram-positive species tested.

**Table 1:** IC_50_ values of gram-positive bacteria.

Extracts	*Staphylococcus aureus*	*Micrococcus luteus*	*Enterococcus faecalis*	*Streptococcus faecalis*	*Staphylococcus epidermis*	*Listeria innocua*
IC_50_ (µg/mL)	57.47 ± 0.005	105.8 ± 0.014	804 ± 0.008	1223 ± 0.006	1892 ± 0.01	≥2000 ± 0.0
Positive control (Ciprofloxacin)	0.28 ± 0.003	2.11 ± 0.01	9.44 ± 0.03	3.56 ± 0.005	0.49 ± 0.002	0.59 ± 0.01

The results are presented as Mean of three independent determinations ± Standard Deviation.

#### 3.1.2 Effect of extract on gram-negative bacteria

**Figure 3:**
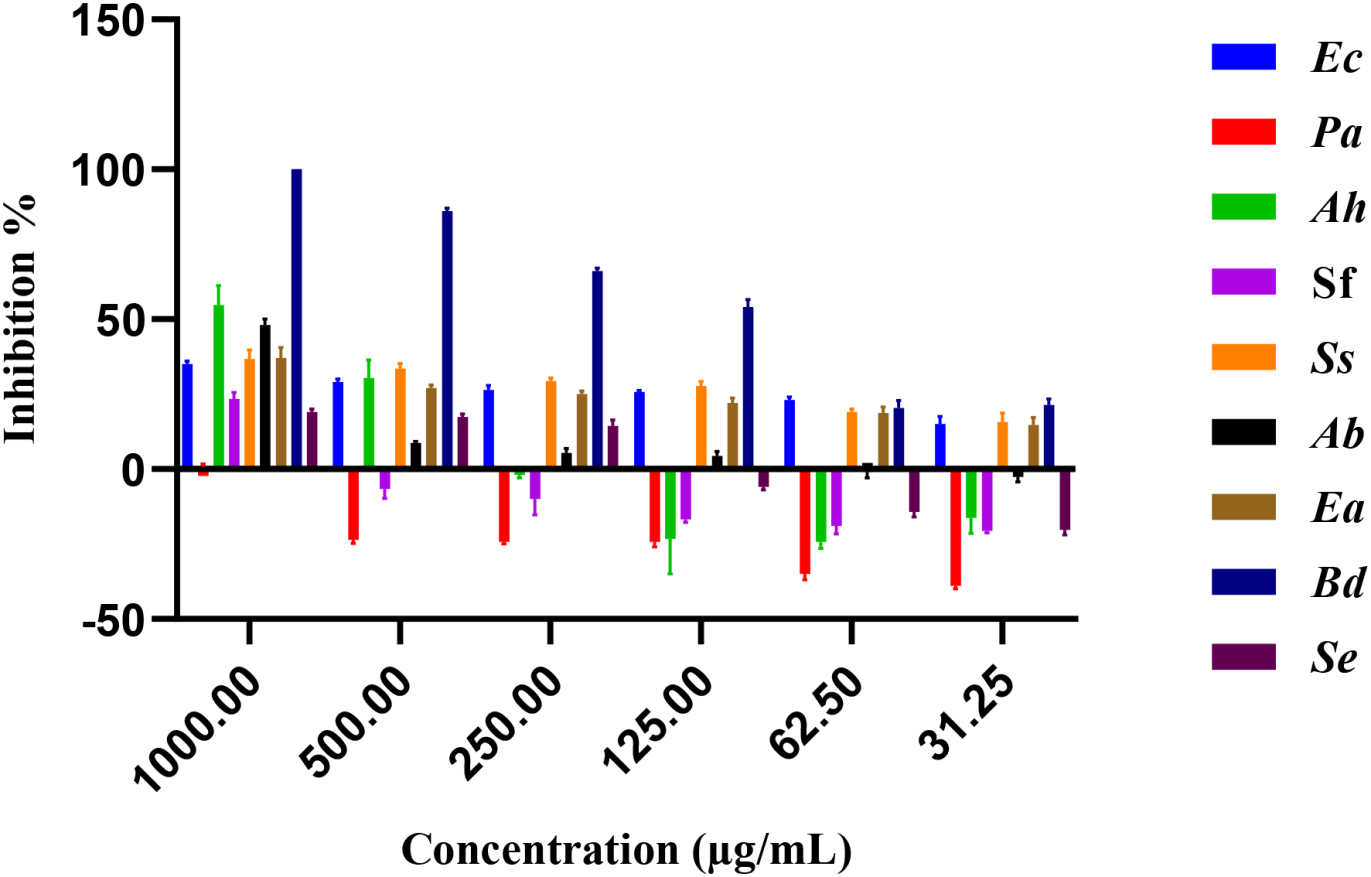
Percentage of inhibition of extract against gram negative bacteria.

**Figure 3** illustrates the inhibitory effects of *E. billardieri* extract on Gram-negative bacteria. The figure displays inhibition percentages across the experimental concentration range (31.25–1000 µg/mL). For strains that did not achieve 50% inhibition within this range, the IC_50_ values reported in **Table 2** are listed as ≥2000 µg/mL, indicating that the actual IC_50_ exceeds the tested concentrations. Compared to Gram-positive species, Gram-negative bacteria exhibited higher IC_50_ values, suggesting lower susceptibility. Among the tested Gram-negative strains, *Brevundimonas diminuta* was the most susceptible, with an IC_50_ value of 127.2 µg/mL. In contrast, *Escherichia coli* and *Pseudomonas aeruginosa* showed the highest IC_50_ values (12,408 µg/mL and 759,196 µg/mL, respectively), indicating significant resistance (**Table 2**). Other species, such as *Aeromonas hydrophila*, *Shigella flexneri*, *Shigella sonnei*, *Acinetobacter baumannii*, and *Enterobacter aerogenes*, exhibited moderate levels of inhibition.

**Table 2:** IC_50_ values for the extract against gram-negative bacteria.

Extracts	*Escherichia coli*	*Pseudomonas aeruginosa*	*Aeromas hydrophila*	*Shigella flexneri*	*Shigella sonnei*	*Acinobacter baumanii*	*Enterobacter aerogenes*	*Brevundimonas diminuta*	*Salmonella enteritidis*
IC_50_ (µg/mL)	≥2000 ± 0.0	≥2000 ± 0.0	866.5 ± 0.009	1047 ± 0.004	≥2000 ± 0.0	1238 ± 0.046	≥2000 ± 0.0	127.2 ± 0.017	≥2000 ± 0.0
Positive control (Ciprofloxacin)	0.02 ± 0.001	0.02 ± 0.002	0.01 ± 0.001	0.02 ± 0.002	0.02 ± 0.001	0.17 ± 0.001	0.04 ± 0.003	2.26 ± 0.03	0.01 ± 0.0

The results are presented as Mean of three independent determinations ± Standard Deviation.

#### 3.1.3 Effect of extract on yeasts

**Figure 4:**
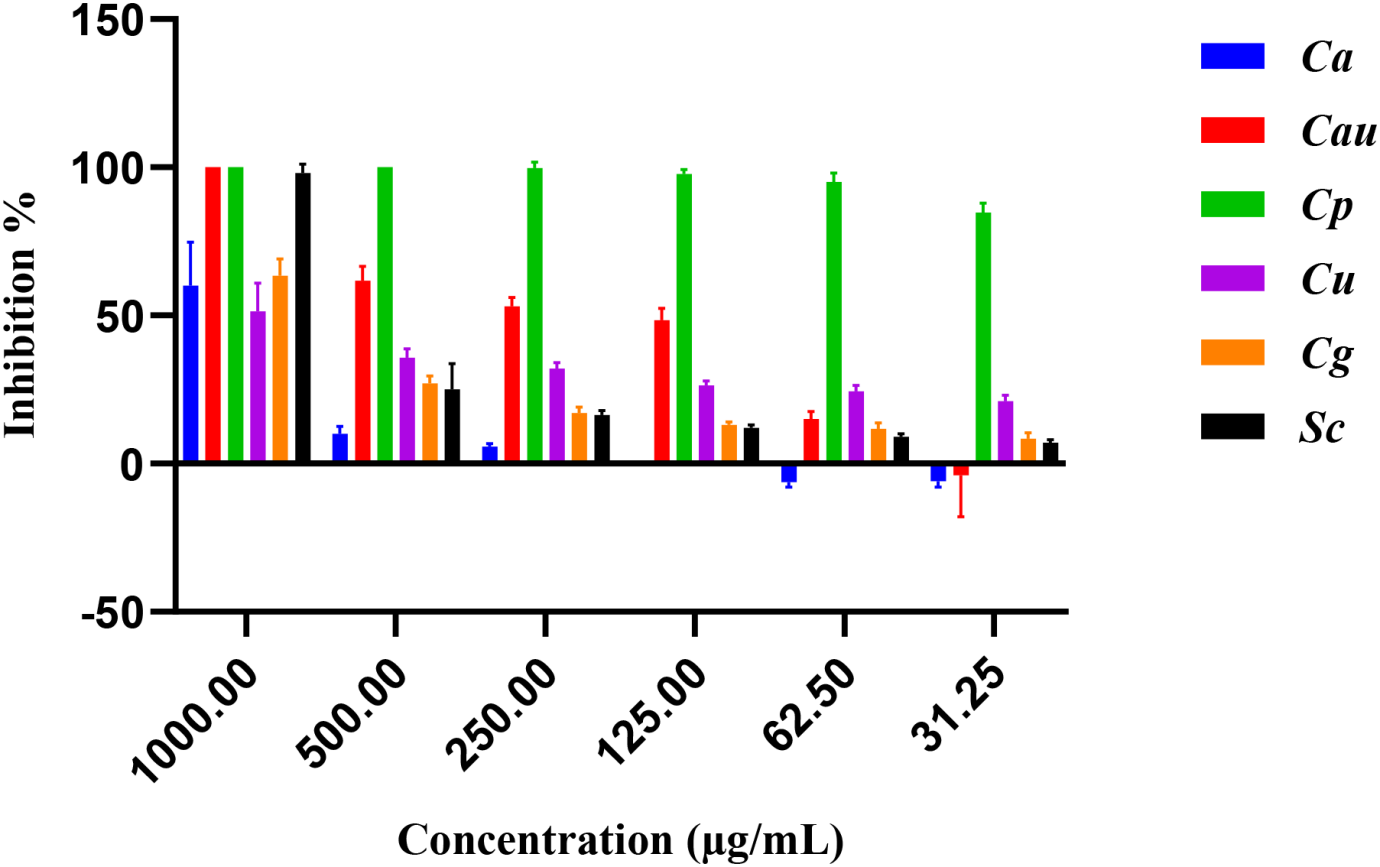
Percentage of inhibition of extract against yeasts.

The antifungal activity of *E. billardieri* extract against yeast species is shown in **Figure 4**. The extract demonstrated substantial effects, with *Candida parapsilosis* and *Candida albicans* being the most susceptible strains, exhibiting IC_50_ values of 11.29 and 63.29 µg/mL, respectively (**Table 3**). Other yeasts, such as *Candida auris* and *Saccharomyces cerevisiae*, displayed moderate susceptibility, with IC_50_ values of 209.3 µg/mL and 594.9 µg/mL, respectively. In contrast, *Candida glabrata*, and *Candida utilis* exhibited relatively higher IC_50_ values (804.5 µg/mL, and 1412 µg/mL, respectively), indicating lower susceptibility.

**Table 3:** IC_50_ values for the extract against yeasts.

Extracts	*Candida albicans*	*Candida auris*	*Candida parapsilosis*	*Candida utilis*	*Candida glabrata*	*Saccharomyces cerevisiae*
IC_50_ (µg/mL)	63.29 ± 0.01	209.3 ± 0.005	11.29 ± 0.05	1412 ± 0.07	804.5 ± 0.01	594.9 ± 0.002
Positive control (Miconazole)	0.01 ± 0.0001	0.10 ± 0.001	0.13 ± 0.002	1.23 ± 0.026	0.12 ± 0.003	0.01 ± 0.0001

The results are presented as Mean of three independent determinations ± Standard Deviation.

#### 3.1.4 Antibiofilm activity

**Figure 5:**
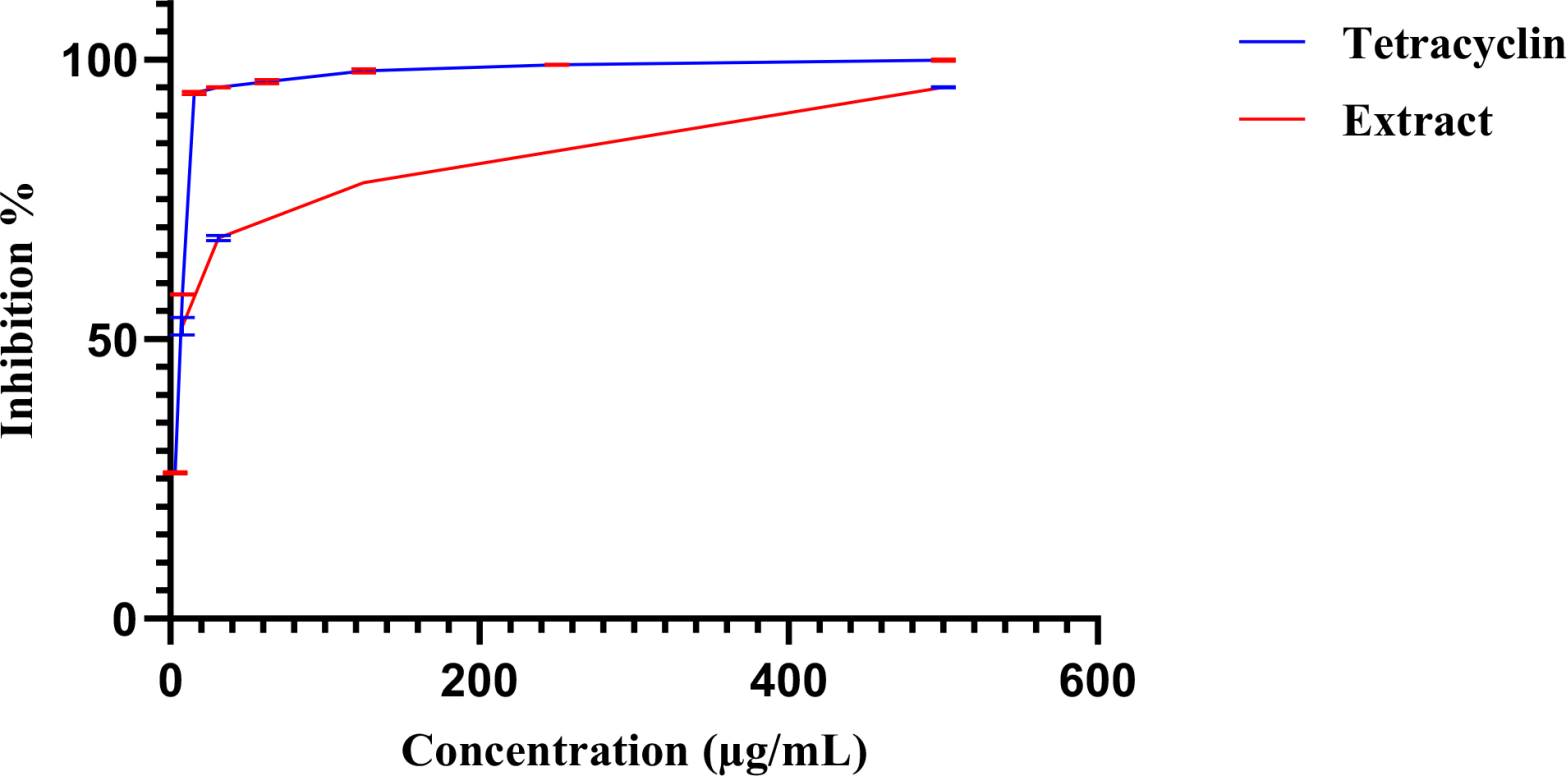
Dose–response curves of biofilm inhibition by the extract (red) and tetracycline (blue). Error bars, shown in the different color as each curve.

The *E. billardieri* extract demonstrated an antibiofilm effect at sub-minimum inhibitory concentrations (sub-MICs). Within 24 hours of biofilm formation, the extract inhibited the growth of all tested strains, achieving an IC_50_ of 6.3 µg/mL (**Figure 5**). However, no significant antibiofilm activity was observed after 48 hours of biofilm formation. These findings suggest the potential use of the extract as an antibacterial agent to inhibit biofilm formation during the early stages.

### 3.2 Anthelminthic activity

**Figure 6:**
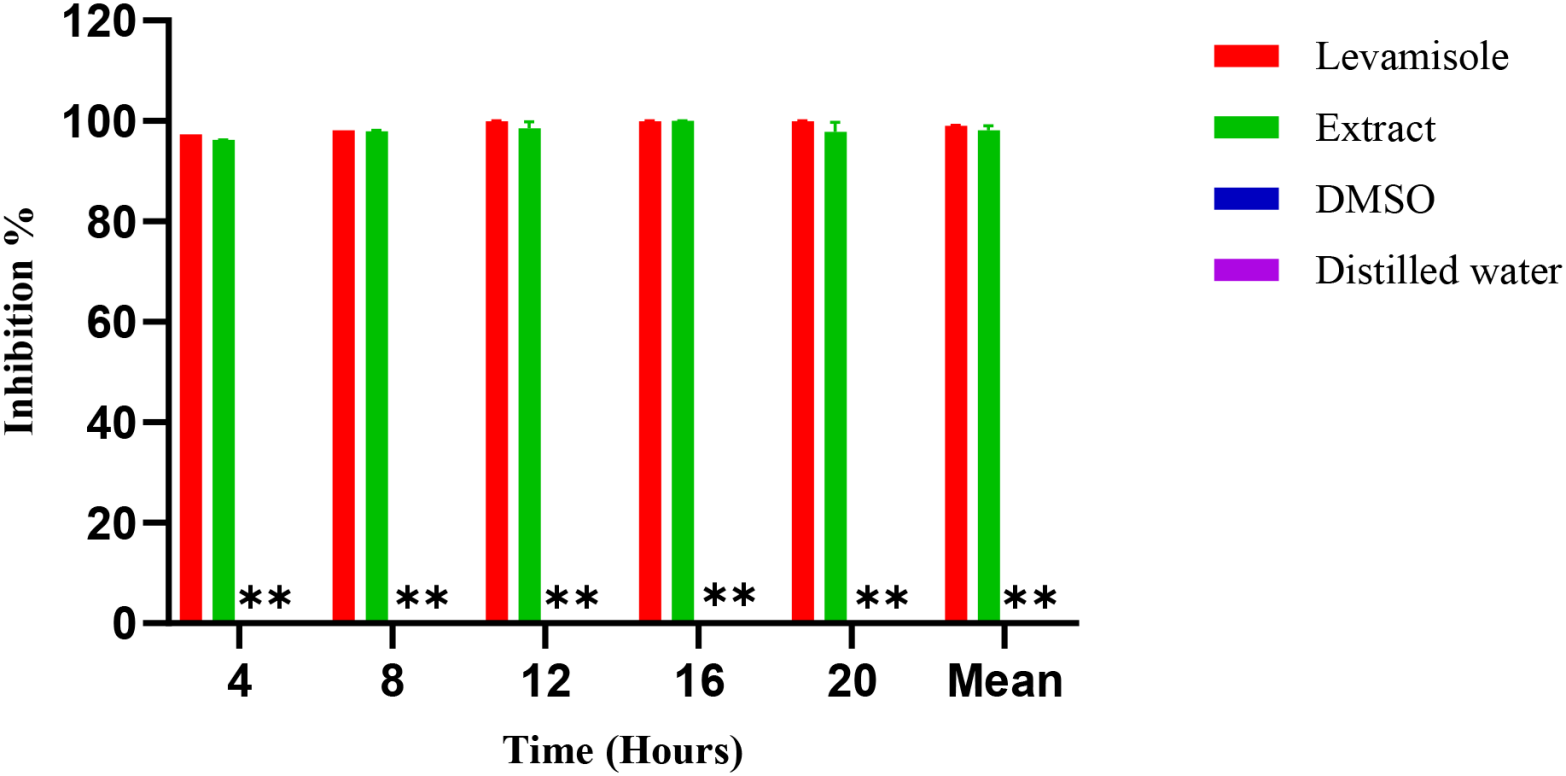
Inhibition (%) over time for Levamisole (positive control), Extract, DMSO, and Distilled water (negative controls). **: DMSO and distilled water show 0% inhibition at all time points and, therefore, appear as baseline bars.

**Figure 7:**
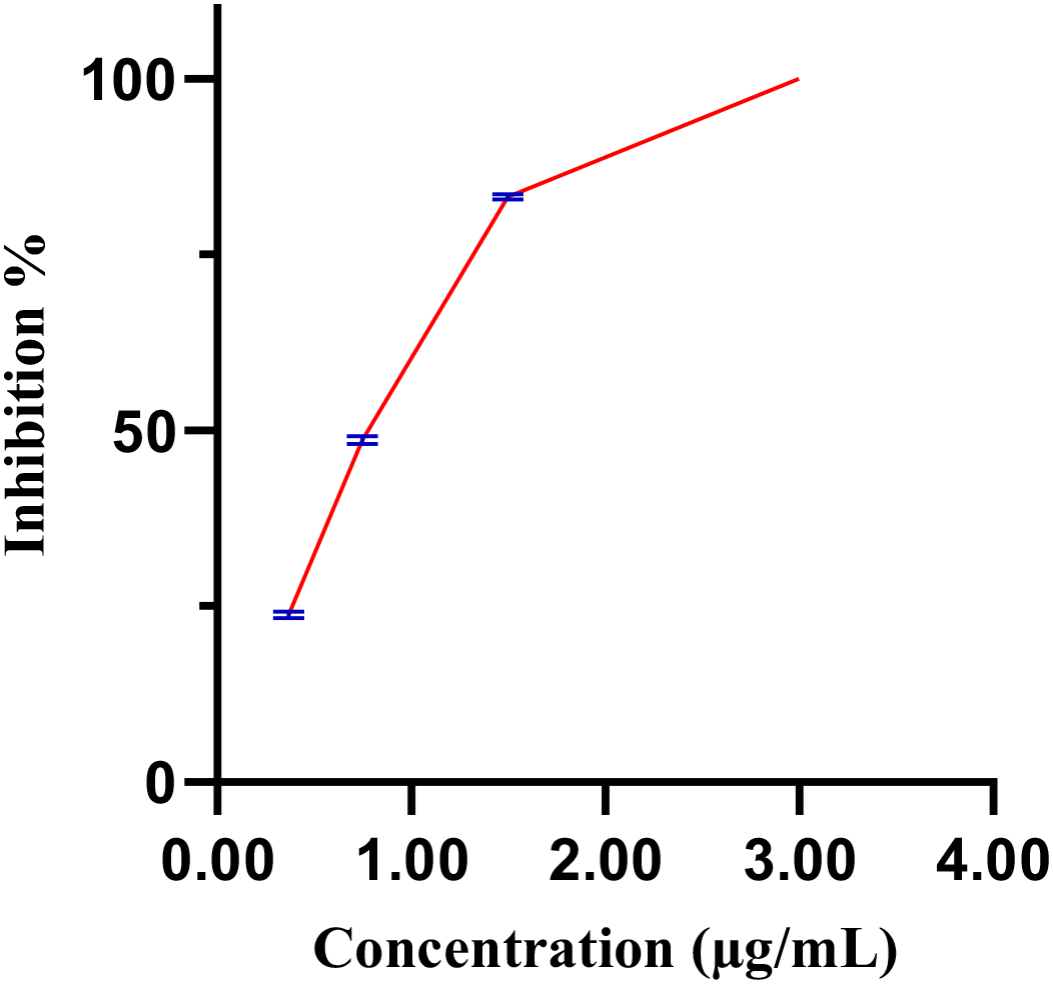
Dose-response curve of the extract, with blue bars indicating standard error.

**Figure 6** illustrates the inhibition percentages of *C. elegance* L4 larval mobility induces by the methanol extract over time. At a concentration of 2.0 µg/mL, the methanol extract completely inhibited larval mobility (97.7 ± 1.5%), closely mirroring the effect of the positive control, levamisole (99.1 ± 1.78%). In contrast, neither 1.5% DMSO nor distilled water (negative control) affected larval mobility. Decreasing the extract concentration from 3 µg/mL to 0.37 µg/mL resulted in a progressive reduction in inhibitory effects, with an estimated IC_50_ of 0.89 µg/mL (**Figure 7**).

### 3.3 Chemical profiling of *E. billardieri*

**Figure 8:**
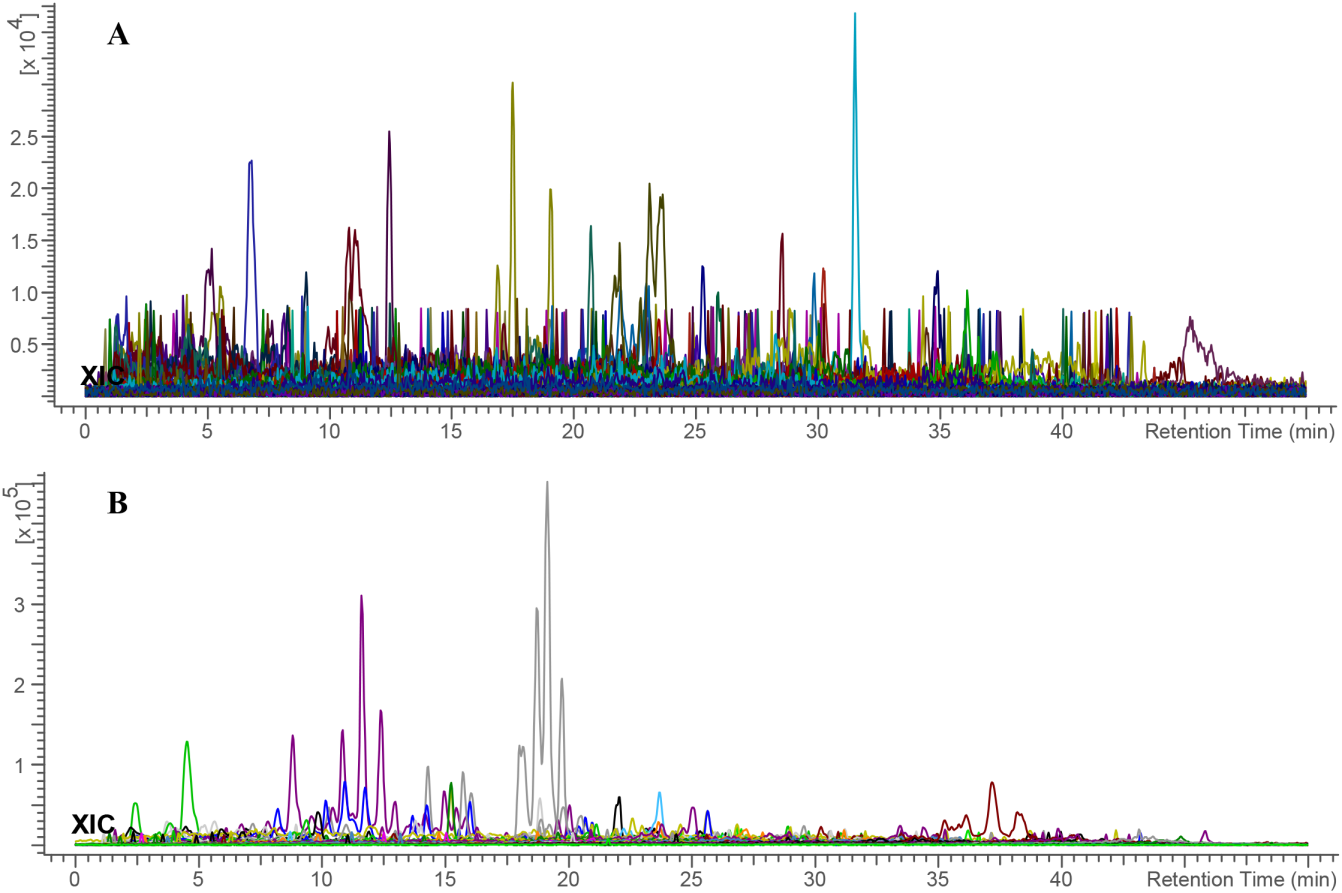
Total ion chromatogram of *E. billardieri* extract in **(A)** positive and **(B)** negative ionization modes. Each color indicates a distinct m/z signal.

The MS data of the *E. billardieri* extract was analyzed and matched against a manually curated database using the ACD/MS Workbook Suite. The total ion chromatogram (TIC) was generated from matched ions, and extracted ion chromatograms (EICs) in both positive and negative ion modes are shown in **Figure 8**. The identified compounds and their fragmentations are listed in **Table 4**. A total of thirty-three components were tentatively identified, comprising seventeen terpenoids, one coumarin, five flavonoids, five phenolic acids, five fatty acids, and four aldehydes. Among these, isorhamnetin-3-O-glucoside and phytolaccagenin exhibited the highest relative contents, each accounting for 11.2% based on peak area comparison. Other major components with contents exceeding 2% included terpinolene, 3,4-dimethoxybenzaldehyde, palmitic acid, thymol, isobornyl formate, isorhamnetin, 1,4-dimethyl-7-(1-methylethenyl)-octahydroazulene, daturadiol, (-)-phyllocladene, eucalyptol, borneol, (1S,4aS,4bR,6aR,8S,10aR,10bR)-8-hydroxy-1-(4-hydroxy-2,2-dimethylbutyl)-4a,4b,7,7,10a-pentamethyl-1,4,5,6,6a,8,9,10,10b,11-decahydrochrysene-2-carboxylic acid.

**Table 4:** MS identification of compounds from *E. billardieri*.

Name	RT (min)	Molecular formula	MW	Area (%)	Application
Isorhamnetin-3-O-glucoside	1.133	C_22_H_22_O_12_	478.1111	11.2%	Treatment of cough, crown heart disease, hyperlipidemia, and angina (Du et al., 2014)
Chlorogenic Acid	1.533	C_16_H_18_O_9_	354.0951	1.4%	Anti-inflammation, anti-oxidation, anti-pathogens, skin diseases, diabetes mellitus, liver and kidney injuries (Nguyen et al., 2024).
Terpinolene	2.467	C_10_H_16_	136.1252	8.2%	Anxiolytic and sedative effects (Del Prado−Audelo et al., 2021)
3,4-Dimethoxybenzaldehyde	3.7	C_9_H_10_O_3_	166.063	3.0%	Antifungal agent (PubChem, n.d.).
Ferulic acid	5.5	C_10_H_10_O_4_	194.0579	0.7%	Antioxidant, antiallergic, hepatoprotective, anticarcinogenic, antibacterial (Kumar et al., 2025).
Palmitic Acid	6.767	C_16_H_32_O_2_	256.2402	5.1%	Used in the production of auxiliary agents for the textile industry or lubricating oils (Dalal et al., 2023).
beta-Caryophyllene Oxide	12.367	C_15_H_24_O	220.1827	1.5%	Often used as a preservative in foods, drugs and cosmetics (Ahmed, 2025).
3-Formyl-4,4,6-trimethylcyclohexa-2,5-dienol	12.633	C_10_H_14_O_2_	166.0994	0.7%	fungicidal properties (PubChem, n.d.).
Thymol	13.133	C_10_H_14_O	150.1045	5.9%	Antimicrobial, antioxidant, anti-inflammatory, cicatrizing activities (Escobar et al., 2020).
Sinapinic acid	14.0	C_11_H_12_O_5_	224.0685	0.2%	Hepatoprotective, cardioprotective, neuroprotective, anti-diabetic, anxiolytic and anti-bacterial activities
beta-Sesquiphellandrene	14.3	C_15_H_24_	204.1878	1.6%	Antirhinoviral, Antiulcer (Joshi et al., 2020).
Isobornyl formate	15.233	C_11_H_18_O_2_	182.1307	5.9%	Antimicrobial and anti-inflammatory effects (Areejit Samal, 2025).
4,4,6a,6b,8a,11,11,14b-Octamethyl-1,2,3,4a,5,6,7,8,9,10,12,12a,14,14a-tetradecahydropicene-3,5-diol	15.7	C_30_H_50_O_2_	442.3811	1.9%	Not available.
Isorhamnetin	17.5	C_16_H_12_O_7_	316.0583	2.9%	Cardiovascular and cerebrovascular protection, anti-tumor, anti-inflammatory, anti-oxidation, organ protection, prevention of obesity (Gong et al., 2020).
1,4-Dimethyl-7-(1-methylethenyl)-octahydroazulene	18.7	C_15_H_24_	204.1878	2.0%	Not available.
Germacrene D	19.133	C_15_H_24_	204.1878	3.7%	Antibacterial activity (Noge and Becerra, 2009).
Daturadiol	19.733	C_30_H_50_O_2_	442.3811	4.6%	Anti-inflammatory (Baig et al., 2021).
Caryophyllene	19.767	C_15_H_24_	204.1878	0.3%	Antioxidant, anti-inflammatory, and anticancer (Scandiffio et al., 2020).
(Z)-4-Dodecenal	20.3	C_12_H_22_O	182.1671	2.1%	Membrane stabilizer, Energy source (Api et al., 2025).
(-)-Phyllocladene	20.767	C_20_H_32_	272.2504	4.7%	Not available.
2,3,6-Trimethylbenzaldehyde	20.967	C_10_H_12_O	148.0888	1.8%	Antimicrobial and anti-acetylcholinesterase effects (Matejić et al., 2018).
p-Coumaric acid	21.9	C_9_H_8_O_3_	164.0473	1.3%	Application in inflammation, cardiovascular diseases, diabetes, and nervous system diseases (Chen et al., 2024).
(1S,4aS,4bR,6aR,8S,10aR,10bR)-8-hydroxy-1-(4-hydroxy-2,2-dimethylbutyl)-4a,4b,7,7,10a-pentamethyl-1,4,5,6,6a,8,9,10,10b,11-decahydrochrysene-2-carboxylic acid	23.7	C_30_H_48_O_4_	472.3553	8.8%	Not available.
Eucalyptol	23.833	C_10_H_18_O	154.1358	2.4%	Anti-inflammatory, antioxidant, antimicrobial, and bronchodilatory effects (Hoch et al., 2023).
Daidzein	25.633	C_15_H_10_O_4_	254.0579	0.9%	Managing conditions like osteoporosis, cardiovascular diseases, and certain cancers (Sun et al., 2016).
Apigenin	26.233	C_15_H_10_O_5_	270.0528	0.9%	Traditional medicine to potential use in cancer therapy, dermatology, and even enhancing reproductive health (Salehi et al., 2019).
Camphor	29.267	C_10_H_16_O	152.1201	0.3%	Antibacterial, antifungal, antioxidant, and anti-inflammatory properties (Del Prado−Audelo et al., 2021).
Phytolaccagenin	34.933	C_31_H_48_O_7_	532.34	11.2%	Antifungal, antihypertensive (Del Prado−Audelo et al., 2021).
Luteolin-7-O-glucoside	36.633	C_21_H_20_O_11_	448.1006	0.7%	Anti-inflammatory, antioxidant, and potential anticancer properties (Caporali et al., 2022).
Borneol	39.267	C_10_H_18_O	154.1358	2.2%	Anti-inflammatory effects, analgesia, antioxidation (Hu et al., 2024b).
Umbelliferone	41.8	C_9_H_6_O_3_	162.0317	0.8%	Anti-inflammatory, antioxidant, antimicrobial, antiviral, and anticancer properties (Mazimba, 2017).
1,4-Dicaffeoylquinic acid	42.2	C_25_H_24_O_12_	516.1268	0.7%	Role in the treatment of respiratory diseases (Hufnagel et al., 2024).
Carotol	43.2	C_15_H_26_O	222.1984	0.2%	Cytotoxic agent against cancer cells, as well as antimicrobial, anti-inflammatory, and mosquito repellent properties (Sharma et al., 2019).

#### 3.3.1 Computer-assisted structure elucidation by MS

The chemical identification process for *E. billardieri* extract was conducted through UHPLC-MS analysis, following a multi-step approach (Hu et al., 2024a). Firstly, precursor ions were automatically calculated using the ACD/MS Workbook Suite. These ultrahigh-resolution mass data were then cross-referenced with chemical databases, including COCONUT (https://coconut.naturalproducts.net/) and a custom database of *Eryngium* plants. The custom database, which contains previously isolated and identified compounds from *Eryngium* species, provide a reliable foundation for correct identification. A variety of compounds were identified from the *E. billardieri* extract, some of which appeared as isomers. For instance, isorhamnetin-3-O-glucoside was identified based on its [M+H]^+^ precursor ion at m/z 479.118 and a retention time of 1.133 minutes. This mass corresponded to the chemical formula C_22_H_22_O_12_, confirming its identification. Experimental fragmentation spectra were then compared to predicted spectra generated by tools such as MS Fragmenter. This comparison refined the list of potential structures, retaining only those with fragmentation patterns closely aligned with the experimental data.

In cases where isomers could not be distinguished by fragmentation patterns alone, retention time calculations were employed. Using ChromGenius, expected retention times were computed and matched with the observed retention times under identical chromatographic conditions. For instance, the identification of chlorogenic acid was confirmed by its retention time of 1.533 minutes and its molecular formula C_16_H_18_O_9._

The combination of fragmentation analysis and retention time prediction enabled the accurate identification of compounds in the *Eryngium* extract. Final confirmations were made by comparing results against reference standards, ensuring robust and reliable identifications. Key compounds identified included ferulic acid, palmitic acid, and beta-caryophyllene oxide. These findings underscore the diverse chemical composition of the *E. billardieri* extract, laying the groundwork for further studies and potential applications.

## 4 Discussions

In our study, we specifically investigated the biological activities of *E. billardieri*, focusing on its antihelmintic, antibacterial, and antifungal properties.

Previous studies have highlighted the significant antioxidant and antimicrobial activities of the ethanolic extract of *E. billardieri* aerial parts, as well as its high phenolic and flavonoid content (Daneshzadeh et al., 2020). Our results align with these findings, demonstrating the extract’s effectiveness against Gram-negative bacteria. Specifically, *E. billardieri* showed significant inhibition against *Staphylococcus aureus* and *Micrococcus luteus*, suggesting that the extract contains compounds active against Gram-positive bacteria. Conversely, the higher IC_50_ values against *Escherichia coli* and *Pseudomonas aeruginosa* indicate a less pronounced effect on Gram-negative bacteria. This could be attributed to the more complex cell wall structures of Gram-negative bacteria, which often hinder the penetration of antimicrobial agents. These findings suggest that *E. billardieri* may have selective antibacterial activity, which warrants further investigation for potential targeted applications.

Biofilm formation by microorganisms is a widespread phenomenon across various ecological niches, and both immunocompetent and immunocompromised individuals are susceptible to *C. albicans* infections. Given their inherent resistance to conventional antifungal treatments, *Candida* biofilms pose a significant clinical challenge (Gulati and Nobile, 2016). While our study did not identify the specific compound responsible for the extract’s antibiofilm activity against *Candida*, the observed IC_50_ value of 6.3 µg/mL is notably low for a crude extract. This is especially surprising given lack of activity against the planktonic form. The antibiofilm effect appears to be transient, as it was present at 24 hours after biofilm formation but diminished after 48 hours. This could be due to chemical instability of the bioactive compounds or their degradation by the tested fungus. Regardless, these results suggest that the extract exerts a fungistatic, rather than fungicidal, effect.

Helminthiasis is a debilitating condition that remains prevalent in many developing countries across Africa, often overlooked due to the majority of research funding being directed toward diseases such as HIV and tuberculosis (Fernandes et al., 2021). This neglected tropical disease (Hotez et al., 2008), caused by various helminths, has shown a concerning increase in drug resistance, highlighting the urgency of addressing this public health issue (Cédric et al., 2023). Our study is the first to demonstrate strong inhibition (IC_50_ of 0.89 µg/mL) in a model nematode, highlighting the potential of *E. billardieri* a natural antihelmintic agent.

Chemical profiling of the extract revealed the presence of several terpenoids, phenolic acids, and flavonoids, including isorhamnetin 3-O-glucoside and phytolaccagenin, which exhibited the highest relative content. These compounds, along with other constituents like terpinolene and thymol, may contribute to the extract’s observed biological activities. However, bioassay-guided purification is needed to pinpoint the specific compounds responsible for each effect.

Our study unveils the promising potential of *E. billardieri* as a source of bioactive compounds for antimicrobial and antihelmintic applications. Future studies should prioritize isolating and characterizing the specific active compounds, elucidating their mechanisms of action, and evaluating their safety and toxicity profiles. Continued research into plant-derived antimicrobials could provide valuable resources for developing novel treatments, especially in the light of the increasing resistance to conventional drugs.

## 5 Conclusion

The methanol extract of *E. billardieri* showed activity against pathogens such as *Staphylococcus aureus, Micrococcus luteus, Candida albicans, and Candida parapsilosis*, as well as *Caenorhabditis elegans*. Although activity was evident, the inhibitory concentrations required were relatively high, indicating a moderate level of potency compared to established antimicrobial agents. Chemical profiling identified diverse range of bioactive compounds, including terpenoids, flavonoids, and phenolic acids, which likely contribute to these observed effects. However, future research should focus on identifying specific components responsible for these activities, further enhancing our understanding of the plant’s therapeutic potential.

## Data Availability

The raw data supporting the conclusions of this article will be made available by the authors, without undue reservation.
